# Optimally Delivered R-da-EPOCH Versus R-CHOP-21 in Primary Mediastinal Large B-Cell Lymphoma: A Real-Life Comparison in a Single Academic Center

**DOI:** 10.3390/cancers17101699

**Published:** 2025-05-19

**Authors:** Alexia Piperidou, Maria K. Angelopoulou, Chrysovalantou Chatzidimitriou, John V. Asimakopoulos, Maria Arapaki, Fotios Panitsas, Gerassimos Tsourouflis, Marina Belia, Iliana Konstantinou, Anastasia Kopsaftopoulou, Athanasios Liaskas, Alexandros Machairas, Maria-Aikaterini Lefaki, Maria Dimitrakoudi, Sotirios Sachanas, Gerassimos A. Pangalis, Konstantinos Konstantopoulos, Eleni Plata, Marina Siakantaris, Theodoros P. Vassilakopoulos

**Affiliations:** 1 Department of Haematology and Bone Marrow Transplantation, National and Kapodistrian University of Athens, Laikon General Hospital, 11527 Athens, Greece; alpiper@med.uoa.gr (A.P.);; 2 Second Propedeutic Department of Surgery, National and Kapodistrian University of Athens, Laikon General Hospital, 11527 Athens, Greece; 3 Department of Haematology, Athens Medical Center, Psychikon Branch, 11525 Athens, Greece

**Keywords:** Primary mediastinal, Large cell lymphoma, B-cell, R-da-EPOCH, R-CHOP-21, RT

## Abstract

Background/Objectives: The National Cancer Institute introduced the intensified R-da-EPOCH regimen in primary mediastinal large B-cell lymphoma (PMLBCL) to improve outcomes while minimizing radiotherapy use. However, there is no randomized comparison of R-da-EPOCH vs. R-CHOP-21. The objective of this study was to compare R-da-EPOCH with R-CHOP-21 in consecutive patients with PMLBCL of a single, large referral center, where the R-da-EPOCH escalation schedule was strictly followed. Methods: We retrospectively analyzed all 35 consecutive patients who received R-da-EPOCH (2017–2022) compared to 35 consecutive patients treated with R-CHOP-21 arm at the same Department, starting from the most recent patient and going backwards (2005–2017). Results: R-da-EPOCH was given strictly in 33/35 (94%) patients. The 5-year freedom from progression (FFP) was 91% vs. 69% (*p* = 0.027). The 5-year event-free survival (EFS) was 84% vs. 69% (*p* = 0.124). The 5-year overall survival (OS) was 97% vs. 80% (*p* = 0.063). Among R-CHOP-21-responders, 20/29 (69%) received RT compared to 2/34 (6%) R-da-EPOCH-responders. In multivariate analysis, R-da-EPOCH remained better than R-CHOP-21 in terms of FFP [hazard ratios (HRs) 0.21–0.26, all *p* < 0.05] and was associated with very favorable HR for EFS and OS. Conclusions: Optimally delivered R-da-EPOCH minimized the use of RT in a real-life setting and provided superior outcomes than R-CHOP-21.

## Simple Summary

R-CHOP + consolidative radiotherapy (RT) has been historically the usual treatment option for PMLBCL with a cure rate of 75–80%. The National Cancer Institute introduced the intensified R-da-EPOCH regimen, which produced better outcomes compared to R-CHOP, while minimizing RT. However, there is no direct randomized comparison of R-da-EPOCH vs. R-CHOP and the majority of the comparative supporting data comes from retrospective series, where the comparisons performed demonstrated only marginally better outcomes. Herein, we report a large, real-life, single-center study aiming to compare R-da-EPOCH with R-CHOP in patients with PMLBCL of a single referral center, where the R-da-EPOCH escalation schedule was strictly followed almost universally. According to our findings, R-da-EPOCH if delivered optimally, eliminates the need for RT and might provide a benefit in disease control and potentially improved survival at the expense of a small, but measurable risk of long-term side effects.

## 1. Introduction

Primary mediastinal large B-cell lymphoma (PMLBCL) is a rare aggressive B-cell lymphoma, recognized as a distinct entity based on clinical, morphologic, immunophenotypic and cytogenetic characteristics [1]. It accounts for approximately 2.5% of non-Hodgkin Lymphomas (NHLs) with an annual incidence rate of 0.4 new cases per million [1,2,3,4,5,6]. PMLBCL typically affects young and middle-aged women, who present, in the majority of cases, with bulky mediastinal lymphadenopathy, which is often mildly or severely symptomatic. Involvement of extrathoracic distal extranodal sites is a rare event at presentation, while bone marrow involvement is extremely rare [7].

First-line treatment is of critical importance in PMLBCL. The efficacy of salvage therapy is often limited, at least prior to the introduction of innovative therapies [8]. Initial therapy includes anthracycline-based chemotherapy combined with rituximab, which has revolutionized the treatment of PMLBCL, producing a significant improvement in disease control and overall survival (OS) [9,10,11,12,13,14]. However, R-CHOP-21 alone is associated with a 20–30% probability of treatment failure and has been traditionally used along with consolidative radiotherapy (RT) in many patients, even in the positron emission tomography (PET) era [12,15,16,17,18,19]. Other, more intensive regimens, such as R-CHOP-14 or R-M(V)ACOP-B, have been used followed by RT or even consolidation with autologous stem cell transplantation (ASCT) but their superiority over R-CHOP-21 is doubtful and has not been tested in randomized trials [13,20,21,22,23,24,25,26,27,28]. The IELSG37 study was suggestive of the potential superiority of more intensive regimens over R-CHOP-21 in terms of a highly reduced rate of a Deauville score of 5 at the end of treatment, but result was not based on the randomized part of the study [25,29].

The long-term consequences of treatment have very rarely been described in PMLBCL, but it is widely known from the experience with Hodgkin Lymphoma (HL) that mediastinal RT is associated with increased risk of secondary malignancies and cardiovascular toxicity, so current research focuses on minimizing RT use [30,31,32,33,34,35,36,37,38].

In order to increase the success rate and reduce the use of RT, the National Cancer Institute (NCI) introduced the intensified R-da-EPOCH regimen, which produced better outcomes than those historically achieved with R-CHOP-21 whilst also minimizing consolidative RT in a phase 2 trial. According to the initially published data, R-da-EPOCH resulted in impressive disease control, with event-free survival (EFS) of 93%, long-term overall survival (OS) rates of 97% at a median follow-up of 63 months and minimal use of RT in only 2/52 patients [39,40]. Following the impressive NCI data, several real-life studies confirmed the efficacy of R-da-EPOCH in the elimination of RT, demonstrating somewhat lower—but still high—disease control rates. However, formal superiority of R-da-EPOCH over R-CHOP-21 has not been clearly demonstrated in retrospective studies [41,42,43,44,45,46,47,48]. Such studies are further compromised by the fact that the R-da-EPOCH regimen is not optimally delivered in a sizeable proportion of patients, since the escalation process is not strictly followed in real-life settings [41,46], while this information was not recorded in detail in other studies [42,44,45,47,48]. Notably, there is no direct randomized comparison of the two protocols except of the preliminary results of an Ukrainian trial, which are in favor of R-da-EPOCH in terms of both disease control and OS [49].

In the Hellenic retrospective comparison, which has not been fully published yet, protocol deviations of variable degrees were recorded in slightly more than 50% of patients [46]. As the reasons of protocol deviations are left to the discretion of the treating physicians and may not be clearly declared in a retrospective setting, it is not scientifically reasonable to compare the optimally treated subgroup of R-da-EPOCH patients with any R-CHOP-21-treated population, because it is impossible to select an appropriate control group. For this reason, we conducted a real-life comparison of R-da-EPOCH versus R-CHOP-21 in patients with PMLBCL treated at a single, large referral academic center, where the R-da-EPOCH escalation schedule was strictly followed in 33/35 patients, i.e., almost universally.

## 2. Patients and Methods

We retrospectively analyzed all 35 consecutive patients who received R-da-EPOCH in the Department of Haematology and Bone Marrow Transplantation of the National and Kapodistrian University of Athens, Laikon General Hospital between 2017 and 2022. A single patient who received R-CHOP-21 in the context of a completely resected mediastinal mass during the same period was excluded. An identical number of 35 consecutive PMLBCL patients served as R-CHOP-21 controls. All 35 patients received R-CHOP-21, since R-CHOP-14 has not been used in our Department for PMLBCL. All patients were treated at the same Department and were selected in a sequential manner, starting from the most recent patient and going backwards (December 2005–2017). As the Department of Haematology was founded in 2008, patients diagnosed between December 2005 and May 2008 were similarly selected by the same database among those treated at the First Department of Internal Medicine of the National and Kapodistrian University of Athens, Laikon General Hospital, which was the “progenitor” of the Department of Haematology with similar referral patterns. Using this strategy of sequential patient analysis to identify the optimal controls, the selection bias was expected to be minimal and potential sources of bias were limited to supportive care and salvage therapy options. Consolidative RT was used at the discretion of the treating physician, usually based on PET/CT results, but was systematically avoided after R-da-EPOCH in case of a Deauville score of 1–4. RT strategies after R-CHOP-21 varied with time with gradual omission of RT in the event of a Deauville score of 1–2 at the end of treatment [15,16,50].

Patients were clinically staged according to Ann-Arbor classification. In accordance with our previous reports, stage IV was defined only in cases where non-contiguous extranodal lymphoma spread was reported [9]. All the remaining cases were considered as “E”. Risk stratification was based on the well-known international prognostic index (IPI) and age-adjusted IPI (aaIPI) [51], as well as on the two prognostic models, which were recently proposed by the Hellenic Lymphoma group (Model A: E/IV and LDH ≥ 2x, Model B: E/IV and bulky disease) [52].

The primary endpoint was freedom from progression (FFP), which was defined as the time interval between treatment initiation and disease progression, relapse, death of therapy- or disease-related procedures without prior progression/relapse or last follow-up. Event-free survival (EFS) was defined as the time interval between treatment initiation and disease progression, relapse, development of acute myeloid leukemia (AML) or HL as secondary primary neoplasms, death of any cause without prior progression/relapse, or last follow-up. OS was defined as the time interval between treatment initiation and death of any cause or last follow-up.

Comparisons between groups were performed by the chi-square test with continuity correction, if required, or the Mann–Whitney test for qualitative and continuous variables, respectively. Survival curves were plotted according to the Kaplan–Meier method and were compared with the log-rank test. Multivariate survival analysis was performed by Cox’s proportional hazards model [53,54,55].

## 3. Results

### 3.1. Patient Characteristics and Delivery of Chemotherapy

Patients’ characteristics in the two groups were absolutely comparable, except for older age in the R-da-EPOCH group (median 35 vs. 28 years, *p* = 0.01; ≥38 years 43% vs. 9%, *p* = 0.001) and more frequent serous effusions in the R-CHOP-21 group (37% vs. 64%, *p* = 0.37) (Table 1). The risk classification of the patients according to the two prognostic models (A and B) [52], as well as the IPI, was also comparable between the two treatment groups (Table 1). R-da-EPOCH was strictly followed in 33/35 patients (94%): 2 patients reached level 4 and 5 but there was no further escalation, despite the lack of prohibitive toxicity. Comparably to the original R-da-EPOCH publication [39], 49% of the 35 patients reached at least level 4, 89% reached at least level 3, and only 6% reached level 1 (Table 2).

### 3.2. Outcomes

Disease progression or relapse was observed in 2 and 11 patients after R-da-EPOCH or R-CHOP-21. Notably, one of the two patients with protocol violation relapsed despite reaching level 4, as she experienced progressive disease at end-of-treatment restaging at roughly 30 days after the last R-da-EPOCH cycle. Two patients developed AML after R-da-EPOCH versus none after R-CHOP-21. Only 1 patient died in the R-da-EPOCH population versus 8/35 treated with R-CHOP-21. All deaths were due to progressive disease.

**Figure 1:**
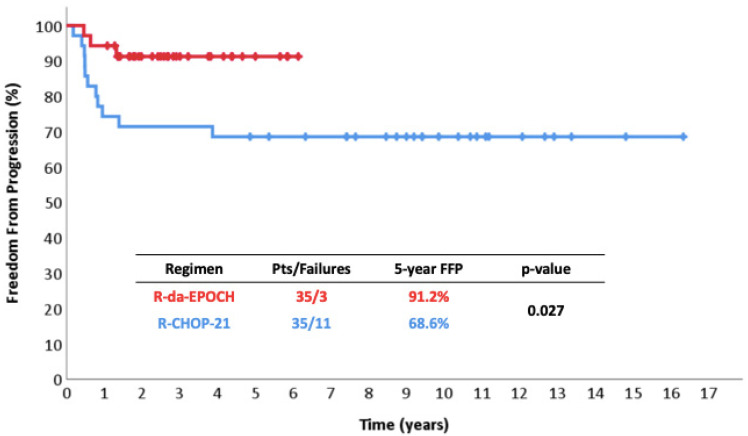
Freedom from progressions in 35 patients with PMLBCL treated with R-da-EPOCH.

**Figure 2:**
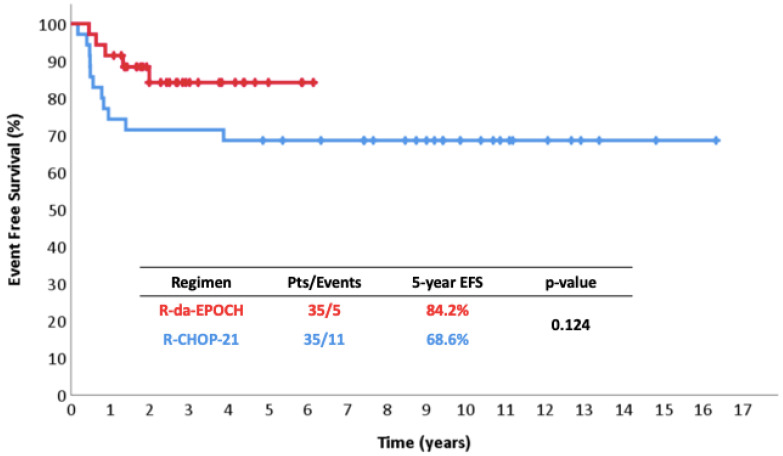
Event free survival in 35 patients with PMLBCL treated with R-da-EPOCH.

**Figure 3:**
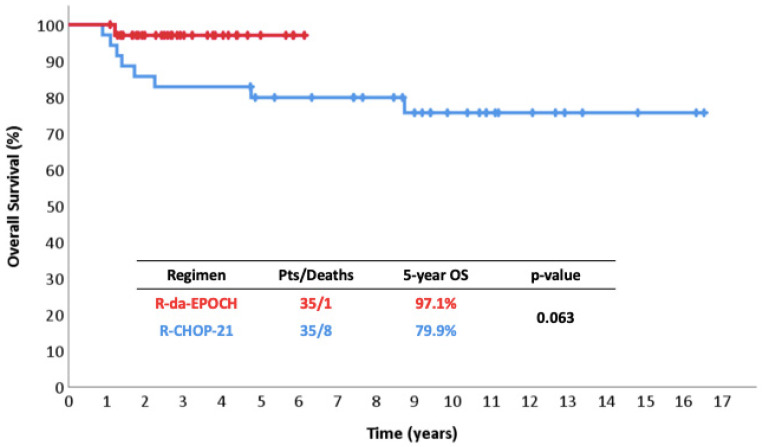
Overall survival in 35 patients with PMLBCL treated with R-da-EPOCH.

The 5-year FFP was 91% vs. 69% (*p* = 0.027) (Figure 1), the 5-year EFS was 84% vs. 69% (*p* = 0.124) (Figure 2), and the 5-year OS was 97% vs. 80% (*p* = 0.063) (Figure 3). Among responders to R-CHOP-21, the majority (20/29, 69%) received RT compared to only 2/34 (6%) R-da-EPOCH responders. Both of the latter had a Deauville score of 5 and conventionally responding disease.

In multivariate analysis -adjusting for age ≥38 years and serositis- R-da-EPOCH remained superior to R-CHOP-21 regarding FFP [hazard ratio (HR) 0.24, 95% confidence intervals (CI) 0.07–0.88, *p* = 0.031]. When the recently published prognostic models (stage E/IV and LDH ≥ 2x and E/IV and bulk) were evaluated in a multivariate model, R-da-EPOCH was again associated with superior FFP rates (HR 0.21–0.26, all *p* < 0.05, Table 3). As shown in Table 3, the difference regarding EFS was borderline (HR 0.37–0.45, *p*-values 0.068 to 0.147), as well as for OS despite very favorable HRs (HR 0.16–0.18, *p* = 0.09–0.12).

## 4. Discussion

During the last decade, R-da-EPOCH has become a very promising intensified regimen with potentially better disease control in patients with PMLBCL compared to R-CHOP-21. Further tothe impressive results of the single-arm phase 2 trial from NCI, the majority of the comparative supporting data comesfrom retrospective series, where the comparisons performed demonstrated numerical improvements but marginal statistical significance, as patient numbers were rather small (Table 4) [39].

In particular, the largest multicenter analysis comparing R-CHOP-21 vs. R-da-EPOCH, which was conducted by Shah et al. and involved 132 patients from 11 US centers, revealed numerical superiority of R-da-EPOCH, without statistical significance for both OS and PFS [OS, HR = 0.63 (0.19–2.15), *p* = 0.46; PFS, HR = 0.62 (0.24–1.47), *p* = 0.28] [41]. Chan et al. conducted a multicenter retrospective study of similar size, which included a smaller R-da-EPOCH-treated population (n = 46) in comparison to 78 patients who received R-CHOP-21 with (n = 37) or without RT (n = 41) versus R-da-EPOCH [47]. The 5-year PFS was 88.5% for R-da-EPOCH compared to 90% for R-CHOP-21 + RT and only 56% for R-CHOP-21 alone (*p* = 0.002). The impact of RT after R-CHOP-21 should be interpreted with caution, as there is a possibility that primary refractory patients were classified in the group of R-CHOP-21 alone, thus underestimating the efficacy of the regimen compared to R-CHOP-21 + RT. Another retrospective study by Malenda et al. in 53 PMLBCL patients from two Polish centers also demonstrated only numerical differences in 12-month PFS (87% vs. 74%, *p* = 0.21) and OS (100% vs. 92%, *p* = 0.81) in favor of the R-da-EPOCH protocol, lacking though of statistical significance, probably due to the low number of patients. In addition, none of the 28 R-da-EPOCH-treated patients proceeded to autoSCT in the frontline setting compared to 9/25 R-CHOP-21-treated patients, who received autoSCT consolidation [48]. One year later, in 2021, Morgenstern et al. conducted a bi-center retrospective analysis of 56 patients with PMLBCL comparing R-da-EPOCH (n = 31) vs. the intensive combination R-CHOP-21/R-ICE (n = 25) and showed similar response rates between treatment groups. Regarding disease control, the investigators showed that an absolute percentage of 16% of the patients treated with R-CHOP-21/R-ICE progressed or relapsed versus 26% of those treated with R-da-EPOCH (*p* = 0.37). The corresponding death rates were 12% versus 16%, respectively (*p* = 0.71) [42].

So far, the only randomized study, which not only numerically but also statistically demonstrated improved outcomes of R-da-EPOCH compared to R-CHOP-21, is a Ukrainian trial based on 84 patients from six centers, the preliminary outcomes of which were presented by Stepanishyna et al. at the 2021 EHA Congress [49]. The study demonstrated a 5-year PFS of 90.9% vs. 64.1% (*p* = 0.002) and a 5-year OS of 97.7% vs. 73.8% (*p* = 0.002) in the R-da-EPOCH and the R-CHOP-21 arm, respectively. However, the complete analysis and the details have not been published yet and the final results have yet to be revealed, whilethere is no reference related to protocol violations. Interestingly, disease control, and especially OS, in the R-CHOP-21 arm appear much worse than expected.

Recently, Rehman et al. published a systematic review and meta-analysis of four studies, including one prospective and three retrospective cohorts [41,42,48,49,56]. A total of 469 patients were included in the analysis, with 256 having received R-EPOCH and 213 R-CHOP-21, respectively. This study demonstrated statistically better disease control with R-da-EPOCH compared to R-CHOP-21 in terms of CR rate (65.5% versus 80.1%, RR = 0.84, 95% CI 0.72–0.99; *p* = 0.04) and marginally PFS (RR = 0.76, 95% CI 0.57–1.02; *p* = 0.06) and, interestingly, an OS benefit (RR = 0.84, 95% CI 0.72–0.97; *p* = 0.02) [56].

The protocol dosing schedule—a fundamental difference in the R-da-EPOCH regimen compared to R-CHOP-21—and, most importantly, its influence on treatment outcomes, are not adequately highlighted in the published comparative analyses. In all the previously reported studies, compliance with the protocol was either not reported at all or was suboptimal, as 38% and 14% of the patients did not undergo dose escalation per protocol in the studies by Shah et al. and Malenda et al. For these reasons, scientifically valid comparisons cannot be drawn. Since, in a single center, R-da-EPOCH is more likely to be delivered in the optimal way and the Department of Haematology of the National and Kapodistrian University of Athens almost strictly follows the guidelines of escalation, the present comparison is the first to show the effect of strict protocol adherence and provides a minimally biased comparison using well-matched subgroups of consecutively treated patients between optimally administered R-da-EPOCH and R-CHOP-21. Despite lack of randomization, the R-da-EPOCH and R-CHOP-21 consecutive patient series were notably similar regarding their baseline characteristics and risk classification, which suggests that the methodology used to select the control group and reduce selection bias was effective.

In the series reported here, R-da-EPOCH offered impressively better disease control, with a long-term FFP increased by an absolute percentage of 22% compared to R-CHOP-21, while minimizing the use of RT in this real-world, single-center context. The majority of R-CHOP-21-treated patients (20/29, 69%) received RT compared to only 2/34 (6%) among R-da-EPOCH-treated patients. One could argue that these 35 sequential controls treated with R-CHOP-21 had a slightly lower-than-expected disease control rate of 69% compared to other series and our previously published data, as the expected rate should be 75–80% [9,10,11,12,17,20,56,57,58,59,60,61], but the method of selection guaranteed the least possible bias. Benefits regarding OS and EFS were statistically ambiguous, probably due to the low number of patients, but the HRs were indeed low in favor of R-da-EPOCH, with an impressive 5-year OS of 97%.

Regarding EFS, the difference was reduced because of the occurrence of two AML cases. Considering the rather small patient sample, the difference in EFS failed to reach statistical significance but still remained numerically high at the level of 15% (Figure 2). Both AML patients are still alive following allogeneic stem cell transplantation. No cases of metachronous classical Hodgkin lymphoma—a well-defined event in PMLBCL—were recorded [62].

The limitations of the present study include the small size of the patient population, which is not so small for a rare disease such as PMLBCL, especially within a single center, and is comparable to many other reports, even multicenter ones. Indeed, it should be noted that the very few previously published comparisons are of the same size in terms of R-da-EPOCH-treated patients [42,47,48] with the exception of the multicenter study by Shah et al., which included 76 patients treated with R-da-EPOCH. Furthermore, unpredictable bias sources may have been present, as this was not a randomized trial. The protocol deviation in 2/35 patients still remains a limitation, but this could only underestimate the superiority of R-da-EPOCH, as 1/2 patients with protocol violation had primary progressive disease. In any case, both patients received a substantial amount of chemotherapy reaching levels 4 and 5. A last potential limitation could be that any OS benefit with R-da-EPOCH might be obscured by the application of novel effective salvage therapies, including checkpoint inhibitors and CAR-T cells [63,64,65,66,67,68,69,70,71,72,73,74,75]. Interestingly, no R-da-EPOCH-treated patients received novel agents in this series compared to one after R-CHOP-21, so this potential limitation is not applicable.

## 5. Conclusions

In conclusion, if delivered optimally, R-da-EPOCH almost eliminates the need for RT and provides benefits in terms of disease control and a meaningful increase in survival, despite the small, but measurable risk of long-term side effects. Thus, optimally delivered R-da-EPOCH appears superior to R-CHOP-21, with much less RT use, but its superiority compared to other intensified regimens still remains unclear. However, its application in the community is suboptimal, so multicenter studies with real-life data and their heterogeneity are still very informative. Reporting on the adherence to the protocol in these studies is also of crucial importance.

## Figures and Tables

**Table 1:** Comparison of patients’ baseline characteristics between R-da-EPOCH and R-CHOP-21 populations.

Patient Characteristics	R-da-EPOCH % n = 35		R-CHOP % n = 35		*p*-Value
	#	%	#	%	
Age (Median, Range)	35 (16–51)		28 (16–59)		0.01
Age ≥ 38 years old	15/35	43	3/35	9	0.001
Females	26/35	74	22/35	63	0.30
Β-symptoms	7/35	20	7/33	21	0.90
PS ≥ 2	7/35	20	8/34	24	0.72
Stage ΙΙΙ/IV	10/35	29	6/33	18	0.31
Any extranodal site	13/35	37	17/35	49	0.33
Extranodal sites ≥ 2	4/35	11	9/35	26	0.12
Extrathoracic disease	8/35	23	4/35	11	0.21
Kidney/Adrenal involvement	3/35	9	2/35	6	0.64
Serous effusions	13/35	37	21/33	64	0.03
Bulky disease ≥ 10 cm	25/35	71	21/32	66	0.61
Serum LDH elevated	29/35	83	30/34	88	0.53
Serum LDH ≥ 2x	15/35	43	11/34	32	0.37
IPI ≥ 2	13/35	37	16/35	46	0.47
aaIPI ≥ 2	13/35	37	12/35	34	0.80
Model A: E/IV and LDH ≥ 2xUNL *					0.89
No adverse factors	15/35	43	15/34	44	
1 adverse factor	12/35	34	10/34	29	
2 adverse factors	8/35	23	9/34	27	
Model B: E/IV and bulk					0.91
No adverse factors	8/35	23	6/32	19	
1 adverse factor	16/35	46	15/32	47	
2 adverse factors	11/35	31	11/32	34	

* UNL: upper normal limit.

**Table 2:** Dose escalation schedule and protocol adherence in R-da-EPOCH-treated patients in comparison to the original NCI study [39].

Maximum R-da-EPOCH Level	All Patients (n = 35)		Strictly Delivered EPOCH Subgroup (n = 33)	
	#	%	#	%
1	2	5.7	2	6.1
2	2	5.7	2	6.1
3	14	40.0	14	42.4
4	9	25.7	8	24.2
5	6	17.1	5	15.2
6	2	5.7	2	6.1
**Maximum R-da-EPOCH level**	**All patients** (n = 35)		**NCI Study [22]** (n = 51)	
	#	%	**#**	**%**
1	2	5.7		6
≥3	31	88.5		
≥4	17	48.5		“>50%”

**Table 3:** Multivariate analysis.

	Freedom from Progression			Event-Free Survival			Overall Survival		
	Hazard Ratio	95% CI	*p*-Value	Hazard Ratio	95% CI	*p*-Value	Hazard Ratio	95% CI	*p*-Value
*Multivariate Model including Treatment Regimen, Age and Presence of any Serositis*									
R-da-EPOCH vs. R-CHOP-21	0.24	0.07–0.88	0.031	0.41	0.14–1.20	0.103	0.16	0.02–1.32	0.089
Age ≥ 38 years old			NS			NS			NS
Any serositis			NS			NS			NS
*Multivariate Model including Treatment Regimen and Prognostic Model A (E/IV and/or LDH ≥ 2x)*									
R-da-EPOCH vs. R-CHOP-21	0.21	0.06–0.76	0.017	0.37	0.12–1.08	0.068	0.17	0.02–1.36	0.095
E/IV and/or LDH ≥ 2xUNL *			0.072			0.069			NS
1 factor vs. 0 factors	4.96	1.26–19.57	0.022	4.75	1.20–18.73	0.026			
2 factors vs. 0 factors	2.66	0.60–11.89	0.20	4.01	1.002–16.03	0.050			
*Multivariate Model including Treatment Regimen and Prognostic Model A (E/IV and/or bulk)*									
R-da-EPOCH vs. R-CHOP-21	0.26	0.07–0.97	0.044	0.45	0.15–1.33	0.147 (NS)	0.18	0.02–1.54	0.12 (NS)
E/IV and/or bulk			NS			NS			NS
1 factor vs. 0 factors									
2 factors vs. 0 factors									

NS: not selected in multivariate analysis; * UNL: upper normal limit

**Table 4:** Published studies comparing R-da-EPOCH vs. R-CHOP-21 in PMLBCL patients.

		Patients (#)		Progression Free Survival (PFS)	Overall Survival (OS)
Author [REF]	Type of Study	R-da-EPOCH Group	Comparator Group	R-da-EPOCH vs. Comparator	R-da-EPOCH vs. Comparator
**Shah et al. [41]**	Propensity-weighted retrospective analysis	76	56 *	HR: 0.62; 95% CI: 0.24–1.47 *p* = 0.28	HR: 0.63; 95% CI 0.19–2.15 *p* = 0.46
**Chan et al. [47]**	Retrospective study	46	78 **	88.5% vs. (90% vs. 56%) ** at 5 years *p* = 0.002	NR
**Malenda et al. [48]**	Retrospective study	28	25 *	87% vs. 74% at 1 year *p* = 0.21	100% vs. 92% at 1 year *p* = 0.81
**Morgenstern et al. [42]**	Retrospective study	31	25 ***	26% vs. 16% relapse at 2 years *p* = 0.37	NR
**Stepanishyna et al. [49]**	Prospective rando-mized clinical trial	44	40 *	90.9% vs. 64.1% at 5 years *p* = 0.002	97.7% vs. 73.8% at 5 years *p* = 0.002
**Rehman et al. [56]**	Systematic review	256	213 ^¶^	RR: 0.76; 95% CI: 0.57–1.02 *p* = 0.06	RR:0.84; 95% CI 0.72–0.97 *p* = 0.02 ^¶¶^
**Piperidou et al.** present study	Retrospective study	35	35 *	91% vs. 69% at 5 years *p* = 0.027	97% vs. 80% at 5 years *p* = 0.063

* R-CHOP-21; ** R-CHOP-21+RT vs. R-CHOP; *** R-CHOP-21/R-ICE; ^¶^ heterogenous; ^¶¶^ OS rates 80.1% vs. 65.5%; NR: not reported.

## Data Availability

The data presented in this study are available on request from the corresponding author due to privacy reasons.
